# SIRT1 Functions as an Important Regulator of Estrogen-Mediated Cardiomyocyte Protection in Angiotensin II-Induced Heart Hypertrophy

**DOI:** 10.1155/2014/713894

**Published:** 2014-12-29

**Authors:** Tao Shen, Ling Ding, Yang Ruan, Weiwei Qin, Yajun Lin, Chao Xi, Yonggang Lu, Lin Dou, Yuping Zhu, Yuan Cao, Yong Man, Yunfei Bian, Shu Wang, Chuanshi Xiao, Jian Li

**Affiliations:** ^1^The Key Laboratory of Geriatrics, Beijing Hospital and Beijing Institute of Geriatrics, Ministry of Health, Beijing 100730, China; ^2^The Second Hospital of Shanxi Medical University, Taiyuan 030001, China; ^3^Department of Cardiology, The General Hospital of Jincheng Anthracite Mining Group Co. Ltd., Jincheng 048006, China; ^4^Capital Medical University Affiliated Beijing Anzhen Hospital, Beijing 100029, China; ^5^College of Life Sciences, Beijing Normal University, Beijing 100875, China; ^6^Department of Cardiology, Shanxi Medical University, Taiyuan, Shanxi 030001, China

## Abstract

*Background*. Sirtuin 1 (SIRT1) is a member of the sirtuin family, which could activate cell survival machinery and has been shown to be protective in regulation of heart function. Here, we determined the mechanism by which SIRT1 regulates Angiotensin II- (AngII-) induced cardiac hypertrophy and injury in vivo and in vitro. *Methods*. We analyzed SIRT1 expression in the hearts of control and AngII-induced mouse hypertrophy. Female C57BL/6 mice were ovariectomized and pretreated with 17*β*-estradiol to measure SIRT1 expression. Protein synthesis, cardiomyocyte surface area analysis, qRT-PCR, TUNEL staining, and Western blot were performed on AngII-induced mouse heart hypertrophy samples and cultured neonatal rat ventricular myocytes (NRVMs) to investigate the function of SIRT1. *Results*. SIRT1 expression was slightly upregulated in AngII-induced mouse heart hypertrophy in vivo and in vitro, accompanied by elevated cardiomyocyte apoptosis. SIRT1 overexpression relieves AngII-induced cardiomyocyte hypertrophy and apoptosis. 17*β*-Estradiol was able to protect cardiomyocytes from AngII-induced injury with a profound upregulation of SIRT1 and activation of AMPK. Moreover, estrogen receptor inhibitor ICI 182,780 and SIRT1 inhibitor niacinamide could block SIRT1's protective effect. *Conclusions*. These results indicate that SIRT1 functions as an important regulator of estrogen-mediated cardiomyocyte protection during AngII-induced heart hypertrophy and injury.

## 1. Introduction

Sirtuins are a highly conserved family of histone/protein deacetylases whose activity can prolong the lifespan of many organisms such as yeast, worms, and flies. In mammalian cells, seven sirtuins (SIRT1-7) could modulate distinct metabolic and stress-response pathways [1, 2]. SIRT1 is mainly located in the nuclei and has been most extensively investigated in the cardiovascular system, which participates in biological functions of energy production, oxidative stress, cell death/survival, and intracellular signaling in hearts. Emerging evidence indicated that SIRT1 might play protective roles in heart injury [3, 4].

Heart remodeling is a process with multifactorial causes. Remodeling hearts present complex phenotypes, including cardiomyocyte hypertrophy, apoptosis, increased fibrosis, and diminished response to stresses. Accumulating evidences have demonstrated that epigenetic modification represents a molecular substrate for cellular stresses, either suppressing or promoting disease initiation [5, 6]. Sirtuins mediate this posttranslational modification by coupling lysine deacetylation to NAD + hydrolysis. SIRT1 is highly expressed in mammalian hearts and regulates many cell functions by deacetylating histones and a number of nonhistone proteins, which is crucially involved in regulation of cardiomyocyte energy metabolism, production of reactive oxygen species, and signaling relevant to cell death/survival [1, 4, 7].

The studies of SIRT1's function in heart hypertrophy are controversial; some data suggested that SIRT1 promoted cardiomyocyte hypertrophy; others indicated that SIRT1 attenuated cardiomyocyte hypertrophy. Resveratrol is wildly used as the endogenous SIRT1 activator [8]. Many of the SIRT1's functions were analyzed based on resveratrol's study. Resveratrol not only activates SIRT1 but also regulates many other transcription factors in cells. In this paper, we discussed our recent findings of the role of SIRT1 in regulating cardiomyocyte hypertrophy and the proposed mechanism behind its protective effects by SIRT1 overexpression and deactivation in cardiomyocytes.

Estrogen signaling is a fundamental process in many different tissue types. E2 is the predominant form of estrogen in nonpregnant females, which binds to both estrogen receptor *α* (ER*α*) and ER*β* in cardiomyocytes. Several groups showed that estrogen (17*β*-estradiol [E2]) prevented hearts from hypertrophy, cell injury, and heart failure [9–12]. In postmenopausal women, myocardial hypertrophy frequently developed faster than that of age-matched men, which could be partially reversed by sex steroid replacement [13–15]. Several studies demonstrated that E2 could attenuate or inhibit the development of heart hypertrophy [14, 15]. However, the molecular pathways that facilitate this process are not completely understood. Therefore, further exploration is required, which may lead to the development of new therapeutic strategies. In the present study, we aimed to systematically investigate the role of E2 in AngII-induced heart hypertrophy and injury.

Here, we provided novel insight into how SIRT1 was involved in estrogen-mediated heart protection effect in Angiotensin II-induced heart hypertrophy and cardiomyocyte apoptosis. We also suggested an improved understanding of the molecular mechanisms by which SIRT1 regulated metabolism and cardiomyocyte apoptosis. We identified SIRT1 as an important regulator in estrogen-mediated heart protection.

## 2. Materials and Methods

### 2.1. Angiotensin II-Induced Heart Hypertrophy Mouse Model

All of the animal experiments conformed to the protocols approved by the Beijing Hospital, Ministry of Health Animal Use and Care Committee, and to the Guide for Care and Use of Laboratory Animals (NIH publication #85-23, revised in 1996).

The 10–12-week-old female C57/BJ6 mice, sham-operated or ovariectomized, were housed in 12 h on/off lighting and fed rodent chow devoid of soy or most plant products. The mice were anesthetized using 1%–1.5% isoflurane in oxygen. Saline or AngII (1.2 mg/kg per day) in saline or saline alone-filled osmotic minipumps (Alzet, Cupertino, CA; DURECT, Cupertino, CA) was provided by 14-day infusion after subcutaneous insertion under inhaled chlorofluorane anesthesia.

At 14 days, the hearts were removed and weighed, and the ratio of heart to total body weight was determined. RNA, DNA, and protein were extracted and the hearts were sectioned for further studies. All mice were identically housed and fed the same chow. The mice were then anesthetized with 3-4% isoflurane and euthanatized by cervical dislocation. The hearts were collected for further analyses.

### 2.2. Mouse Ovariectomy (Ovx) and 17*β*-Estradiol Administration

The female C57BL/6 mice (18–22 g, 8-week-old) were randomly allocated into three groups (*n* = 8–10 per group). All of the animals were anesthetized with 1%–1.5% isoflurane and were ovariectomized via the dorsal route. Sham-operated mice were used as a control. The control and Ovx groups received sesame oil as a placebo 2 weeks after the Ovx. Two weeks after the ovariectomy, the Ovx + AngII + E2 group received an E2 injection 3 days before AngII injection and 14 more consecutive days after AngII injection at a 17-*β*-estradiol dosage of 0.20 mg/day/kg. This estradiol dosage was shown to yield normal uterus/body weight mice in Ovx group. After the induction, the mice were anesthetized with isoflurane and euthanatized by cervical dislocation, and the hearts were collected for further analysis. The ratio of the uterine weight to the body weight was calculated to verify the ovariectomy and E2 replacement models [9, 16, 17].

### 2.3. Adenovirus Construction

Recombinant adenoviruses containing mouse SIRT1 cDNA (SIRT1-Adv) were prepared using the AdEasy system according to the manufacturer's protocol. The *β*-gal vector adenovirus (Vector-Adv) was used as the control in this study. The SIRT1-Adv infection was performed at a multiplicity of infection (m.o.i.) of 30. Vector-Adv was added to the control groups to maintain a consistent viral load [9].

### 2.4. Isolation, Culture, and Adenoviral Infection of Rat Cardiac Myocytes

Neonatal rat ventricular myocytes (NRVMs) were isolated from 1-day-old Sprague Dawley rats via trypsin and collagenase type II combined digestion. The cardiomyocytes were plated at a density of 6.6 × 10^4^ cells/cm^2^ in phenol red-free high glucose DMEM supplemented with 10% FBS in the presence of 0.1 mM 5-bromo-2-deoxyuridine. The adenovirus-mediated gene transfer was performed following 16 h of quiescence in serum and phenol red-free DMEM for 24 h and then treated with 100 nM AngII for another 24 h [9].

### 2.5. Measurement of Protein Synthesis

After 24 h in media without serum, the NRVMs were treated with 100 nM AngII in the presence or absence of 1 × 10^−8^ M E2 or estrogen antagonist ICI 182,780 (Fulvestrant). The NRVM hypertrophy was measured by protein and DNA ratio.

### 2.6. H&E Staining and Cell Size Analysis

Histology assays were performed on adult hearts and sections as previously described [9]. Cultured cell surface area was determined under the various conditions after culturing on cover slips. The tissues or cells were stained by H&E staining kit according to the manufacturer's protocol (Sigma-Aldrich). Cardiomyocyte surface area was quantified by imaging to the complete boundary of 100–200 individual cells/condition for all samples using ImageJ software (http://rsb.info.nih.gov/ij/).

### 2.7. In Situ Detection of Reactive Oxygen Species (ROS)

To evaluate vascular ROS production in situ, frozen, unfixed, whole heart cross sections or cultured NRVMs were stained with 10 *μ*M DHE (Sigma) for 30 min in a dark, humidified chamber at 37°C. ROS generation was indicated by red fluorescence and was visualized with fluorescence microscopy and qualified by ImageJ software [9].

### 2.8. Real-Time RT-PCR

Real-time RT-PCR was performed using the iCycler iQ system (Bio-Rad) and a reaction mixture containing iQ SYBR Green real-time RT-PCR supermix (Bio-Rad) according to the manufacturer's protocol. Briefly, total RNA was extracted using Tri Reagent (Sigma-Aldrich). One microgram of RNA was then reverse-transcribed into first-strand cDNA using random primers and Moloney murine leukemia virus reverse transcriptase (Promega) following the manufacturer's protocol. The PCR profile was as follows: 95°C for 30 seconds and 40 cycles of 95°C for 5 seconds and 60°C for 34 seconds. The amount of SYBR Green was measured at the end of each cycle. The cycle number at which the emission intensity of the sample rose above the baseline was referred to as the threshold cycle and was proportional to the target concentration. The data presented are the average of 3–5 independent experiments. The primers used were as follows:

18S RNA 5′ GGAAGGGCACCACCAGGAGT,

18S RNA 3′ TGCAGCCCCGGACATCTAAG,

ANF 5′ GATAGATGAAGGCAGGAAGCCGC,

ANF 3′ AGGATTGGAGCCCAGAGTGGACTAGG,

*β*-MHC 5′ TGCAAAGGCTCCAGGTCTGAGGGC,

*β*-MHC 3′ GCCAACACCAACCTGTCCAAGTTC,

SIRT1 5′ AGTTCCAGCCGTCTCTGTGT,

SIRT1 3′ GATCCTTTGGATTCCTGCAA.

### 2.9. Western Blot

Western blot was used to assess SIRT1 protein abundance using a primary antibody directed against SIRT1 (Millipore). In a subset of the experiments, adenosine monophosphate- (AMP-) activated protein kinase (AMPK), phospho-AMPK, and cleaved caspase-3 (Cell Signaling Technology) were measured with the corresponding antibodies. GAPDH antibody (Sigma-Aldrich) was used for normalization. ImageJ software (NIH) was used to perform densitometric analysis [9].

### 2.10. Terminal Deoxynucleotidyl Transferase-Mediated dUTP Nick End Labeling (TUNEL) in Cultured Cardiac Myocytes

Nuclear fragmentation was detected by TUNEL staining with an in situ cell death detection kit (Roche). The nucleuses were counterstained by Hoechst 33342. Cells (500–700) in 10 randomly chosen fields from each tissue section/cultured cell slide were counted to determine the percentage of apoptotic nuclei. Each data point indicated the results from 1600 to 2000 cells from 4 independent experiments.

## 3. Statistical Analysis

**Figure 1:**
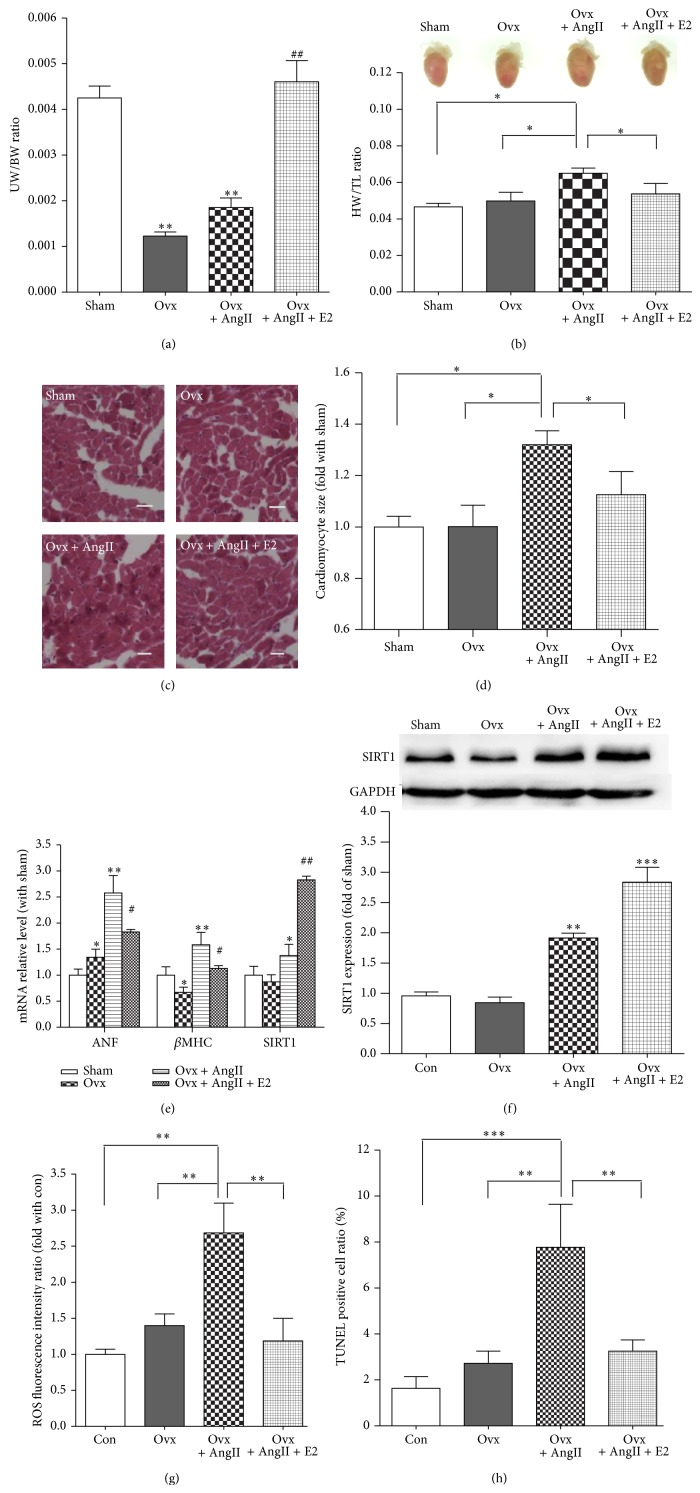
17*β*-Estradiol (E2) elevates endogenous SIRT1 expression and attenuates Angiotensin II-induced heart hypertrophy. (a) The uterus weight and body weight ratio in mice that were sham-operated (sham), ovariectomized (Ovx), ovariectomized with Angiotensin II (AngII) infusion (Ovx + AngII), or ovariectomized with E2 replacement and AngII infusion (Ovx + AngII + E2) in vivo (*n* = 6, ^**^ *P* < 0.01 versus sham, ^##^ *P* < 0.01 versus Ovx or Ovx + AngII group). (b) Mouse heart samples (5x), heart weight, and tibia length ratio of sham, Ovx, Ovx + AngII, and Ovx + AngII + E2 mice (*n* = 6, ^*^ *P* < 0.05, ^**^ *P* < 0.01). (c)-(d) H&E staining of mouse heart samples and cardiomyocyte size statistical analysis (*n* = 5, magnification bar = 100 μm). (e) ANF, *β*-MHC, and SIRT1 mRNA expression in mouse heart as assayed by real-time PCR (*n* = 6, ^*^ *P* < 0.05 versus sham, ^**^ *P* < 0.01 versus sham, ^#^ *P* < 0.05 versus Ovx + AngII, and ^##^ *P* < 0.01 versus Ovx + AngII). (f) SIRT1 expression in mouse heart as assayed by Western blot and the averaged data (*n* = 6, ^**^ *P* < 0.01 versus sham, ^***^ *P* < 0.001 versus sham). (g) The average data of ROS fluorescence intensity relative ratio in mouse heart analyzed by DHE staining (*n* = 5-6, ^**^ *P* < 0.01). (h) The average data from TUNEL staining of mouse heart samples (*n* = 5-6, ^**^ *P* < 0.01, ^***^ *P* < 0.01).

**Figure 2:**
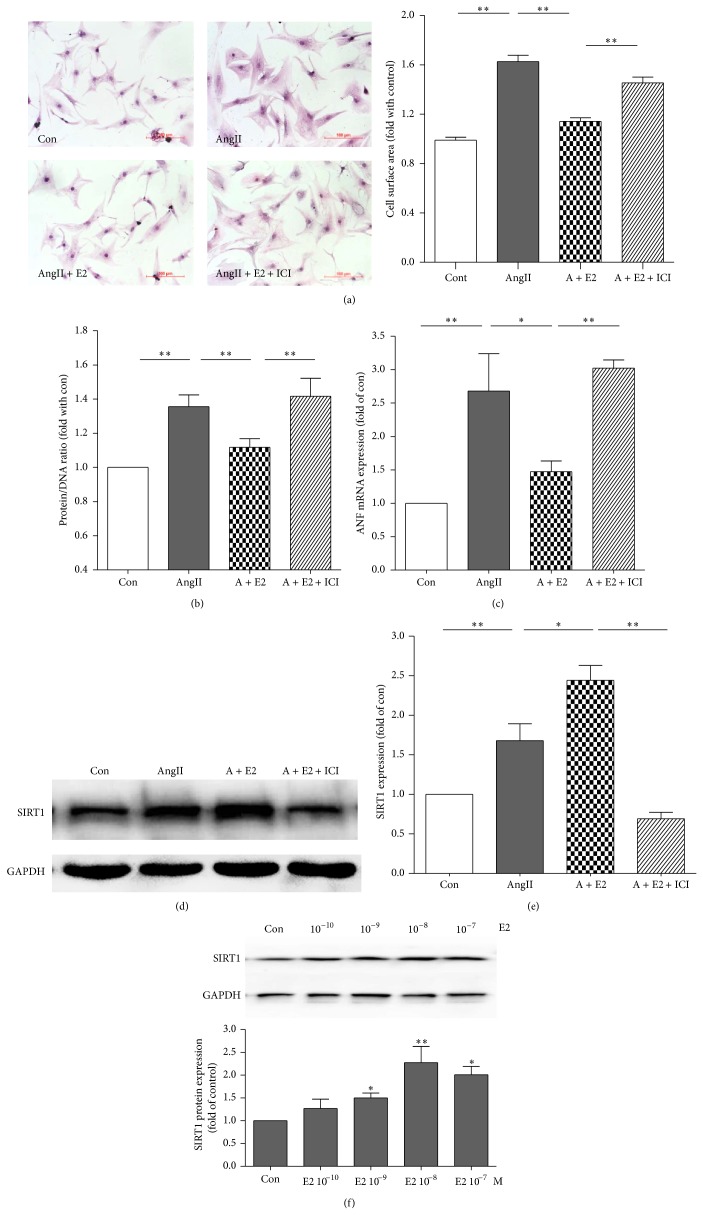
Estrogen upregulates endogenous SIRT1 gene expression. (a) Representative H&E staining and the average cell surface area of NRVMs in control (Con), AngII, AngII + E2, and AngII + E2 + ICI (*n* = 5, ^**^ *P* < 0.01). (b)-(c) The average data of protein and DNA relative ratio (b) and ANF mRNA expression (c) in con, AngII, AngII + E2, and AngII + E2 + ICI groups of NRVMs (*n* = 4, ^*^ *P* < 0.05, ^**^ *P* < 0.01). (d)-(e) SIRT1 expression in the 4 groups of NRVMs, as assayed by Western blot and the average data (*n* = 4, ^*^ *P* < 0.05, ^**^ *P* < 0.01). (f) SIRT1 protein expression (assayed by Western blot) in the NRVMs treated with 1 × 10^−10^ M, 1 × 10^−9^ M, 1 × 10^−8^ M, and 1 × 10^−7^ M of E2 for 24 h (*n* = 3, ^*^ *P* < 0.05 versus control, ^**^ *P* < 0.01 versus the control).

**Figure 3:**
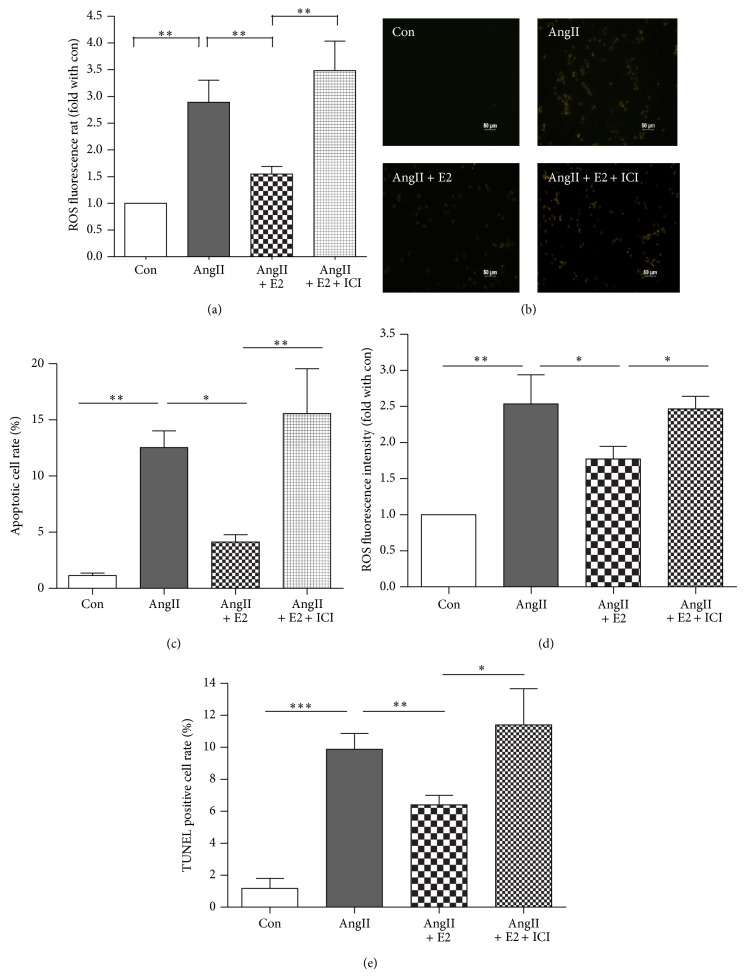
Estrogen attenuates Angiotensin II-induced oxidative stress and cell apoptosis. (a) The average data of ROS fluorescence intensity relative ratio in the NRVMs of Con, AngII, AngII + E2, and AngII + E2 + ICI by DHE staining (*n* = 5-6, ^**^ *P* < 0.01). (b)-(c) Representative TUNEL staining and the average data of the NRVMs of the 4 groups of NRVMs (*n* = 3, 8 *P* < 0.05, ^**^ *P* < 0.01). (d) The average data of ROS fluorescence intensity relative ratio in the 4 groups of NRVMs assayed by DHE staining (*n* = 4, 8 *P* < 0.05, ^**^ *P* < 0.01). (e) The average data of TUNEL positive ratio of Con, AngII, AngII + E2, and AngII + E2 + Nic NRVMs (*n* = 4, ^*^ *P* < 0.05, ^**^ *P* < 0.01, ^***^ *P* < 0.001).

**Figure 4:**
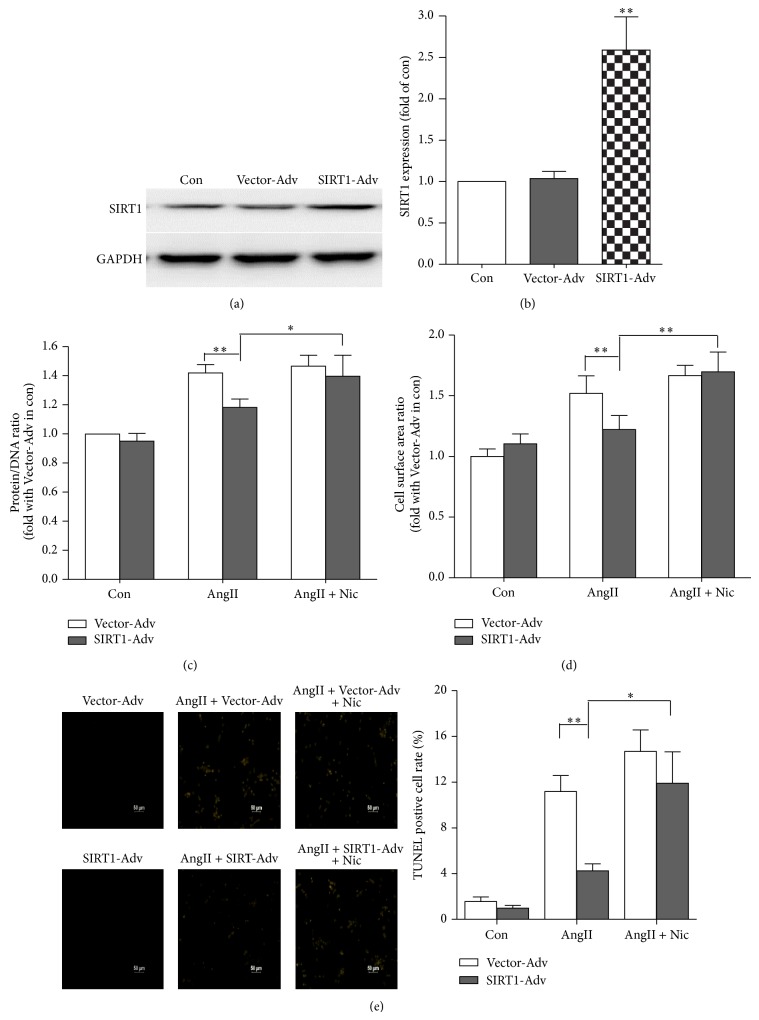
SIRT1 overexpression blocks cardiomyocyte hypertrophy and oxidative stress-induced apoptosis in cardiomyocytes. (a)-(b) Western blot of SIRT1 protein expression and the average data of 30 m.o.i. of control vector adenovirus (Vector-Ad) and 30 m.o.i. of SIRT1 adenovirus infected NRVMs (*n* = 3, ^**^ *P* < 0.01). (c)–(e) The average data of protein and DNA relative ratio (c), the average data of cell surface area (d), and TUNEL staining average data (e) in the NRVMs infected by Vector-Ad or SIRT1-Ad and treated with AngII or AngII + niacinamide (NIC) (*n* = 4, ^*^ *P* < 0.05, ^**^ *P* < 0.01).

**Figure 5:**
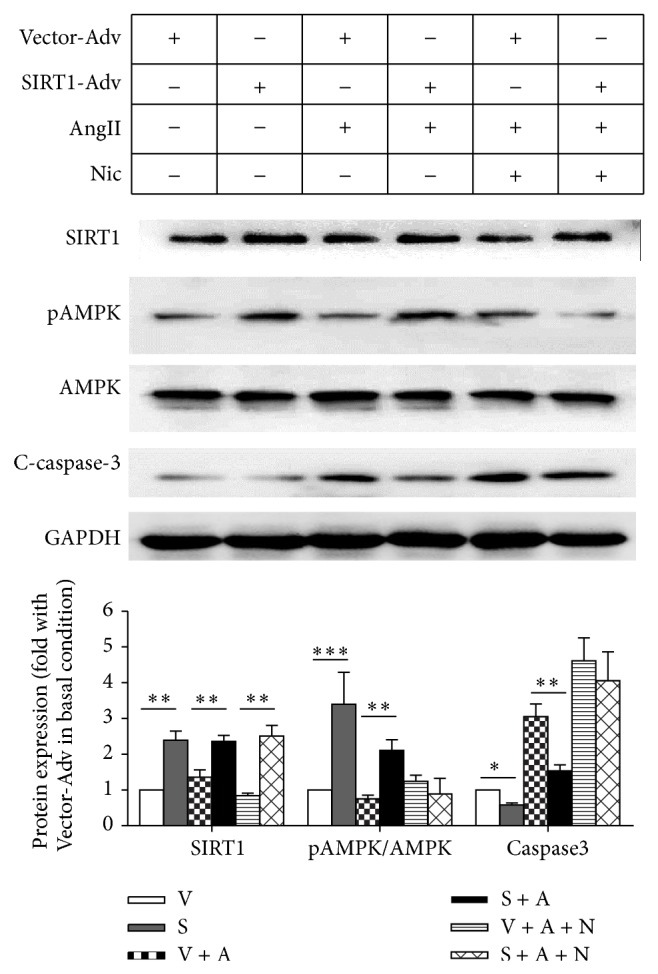
SIRT1 participates in MAPK phosphorylation and cell apoptosis signal regulation.Western blot and average data of SIRT1, p-AMPK, AMPK, cleaved caspase-3, and GAPDH in the NRVMs treated by ethanol (dissolvent control group), 17*β*-estradiol, AngII, or AngII + ICI182,780 (ethanol: EtOH; 17*β*-estradiol: E2; ethanol + AngII: EtOH + A; E2 + AngII; ethanol + AngII + ICI; E2 + AngII + ICI; *n* = 3, ^*^ *P* < 0.05, ^**^ *P* < 0.01, and ^***^ *P* < 0.001).

All values are represented as the mean ± S.E. of the indicated number of measurements. The nonparametric Mann-Whitney test was used to determine statistical differences between two mice groups. A one-way analysis of variance test for post hoc multiple comparison was used to determine significance in Figures 1, 2, 3, and 4(b). Two-way ANOVA was used to examine the differences of vector adenovirus and SIRT1 adenovirus in the (1) control condition, (2) AngII treated conditions, and (3) AngII and niacinamide treated conditions (Figures 4(c), 4(d), 4(e), and 5). Probability values of less than 0.05 were considered significant.

## 4. Results

### 4.1. 17*β*-Estradiol (E2) Elevates Endogenous SIRT1 Expression and Attenuates Angiotensin II-Induced Heart Hypertrophy In Vivo

To explore the protective effect of 17*β*-estradiol in heart hypertrophy, we used an AngII-induced heart hypertrophy mouse model. We performed ovariectomy (Ovx) in the female C57BL/6 mice. Sham-operated mice were used as a control. Two weeks after the ovariectomy, the sham group and Ovx group received sesame oil as placebo and saline-filled osmotic minipumps (Alzet, Cupertino, CA) were provided by 14-day infusion after subcutaneous insertion, Ovx + AngII received AngII (1.2 mg/kg per day) in saline-filled osmotic minipumps, and Ovx + AngII + E2 group received AngII (1.2 mg/kg per day) in saline-filled osmotic minipumps and E2 injection once each day for 3 days before AngII injection and 14 more consecutive days after AngII injection at a dosage of 0.20 mg/day/kg. This estradiol dosage was shown to yield normal uterus/body weight mice after ovariectomy (Figure 1(a)).

After AngII infusion for 2 weeks, the heart weight and tibia length ratio (Figure 1(b)) and cardiomyocyte size (Figures 1(c)-1(d)) were increased 39.65% and 32.00%, respectively, in the Ovx + AngII group compared with sham group, and E2 could partially reverse this phenotype. In addition, ANF and *β*MHC, the markers of heart hypertrophy, were elevated in Ovx + AngII group. E2 could reverse ANF and *β*MHC elevation, too (Figure 1(e)). Moreover, SIRT1 in Ovx group was slightly downregulated (mRNA 0.91 ± 0.25-fold and protein 0.87 ± 0.30-fold compared with sham group) and increased in the Ovx + AngII group (mRNA 1.37 ± 0.47-fold and protein 1.79 ± 0.10-fold compared with sham group). This slight increase was enhanced dramatically in Ovx + AngII + E2 group of mRNA to 2.83 ± 0.16-fold and protein to 2.74 ± 0.53-fold (Figures 1(e) and 1(f)). In addition, AngII could induce heart oxidative stress and cardiomyocyte apoptosis, which could be attenuated by E2 treatment, as shown by DHE and TUNEL staining (Figures 1(g) and 1(h)). These in vivo data suggest that SIRT1 was involved in heart hypertrophy development and was correlated with E2's protection effect in hearts.

### 4.2. Estrogen Upregulates Endogenous SIRT1 Gene Expression in NRVMs

In the present study, we found that 1 × 10^−8^ M E2 could effectively attenuate AngII-induced hypertrophy, whereas E2 inhibitor ICI blocked this protective effect in cultured NRVMs, as analyzed by cell surface analysis, protein/DNA ratio, and ANF mRNA expression (Figures 2(a)–2(c)). AngII could slightly upregulate SIRT1 expression; E2 could further increase SIRT1 expression to a large extent in AngII group, which could be inhibited by E2 inhibitor ICI in cultured NRVMs (Figures 2(d)-2(e)). We also found that E2 enhanced SIRT1 expression in a concentration-dependent manner. E2 upregulated SIRT1 expression, and 1 × 10^−8^ M E2 increased SIRT1 expression to its peak level (2.27 ± 0.5-fold) (Figure 2(f)). Therefore, we used 1 × 10^−8^ M E2 in the subsequent experiments.

### 4.3. Estrogen Attenuates Angiotensin II-Induced Oxidative Stress and Cell Apoptosis

E2 also attenuated AngII-induced oxidative stress in NRVM, as shown by DHE staining (Figure 3(a)) and apoptosis as assayed by TUNEL staining (Figures 3(b), 3(c), 3(d), and 3(e)). Taken together, these results suggest that E2 could suppress cardiomyocyte oxidative stress and apoptosis.

### 4.4. SIRT1 Overexpression Blocks Cardiomyocyte Hypertrophy, Oxidative Stress, and Apoptosis

To determine whether SIRT1 upregulation is sufficient to protect cardiomyocyte function, SIRT1 was overexpressed in NRVMs using adenoviral vectors. Figures 4(a)-4(b) illustrate that SIRT1 adenovirus (SIRT1-Ad) but not vector adenovirus (Vector-Ad, as a vector adenovirus control) substantially increased SIRT1 protein abundance. Furthermore, protein/DNA ratio assay (Figure 4(c)) and cell surface area assay (Figure 4(d)) indicated that SIRIT1 overexpression could partially block cardiomyocyte hypertrophy. TUNEL assay also revealed that SIRT1 overexpression increased the cell viability compared to the control group (Figure 4(e)). However, SIRT1 inhibitor niacinamide could reverse these protective effects. These results indicated that SIRT1 overexpression could block AngII-induced ROS production, cardiomyocyte hypertrophy, and apoptosis.

### 4.5. SIRT1 Participates in AMPK Phosphorylation and Cell Apoptosis Signal Regulation

Our results indicate that E2 could reduce the AngII-induced cell hypertrophy by upregulation of AMPK phosphorylation and SIRT1 expression. Because AMPK plays a significant role in cardiomyocyte hypertrophy, we examined the effect of E2 on AMPK activation. A Western blot analysis demonstrated that E2 increased AMPK phosphorylation in normal and AngII stimulation conditions to 3.40 ± 1.28-fold and 2.11 ± 0.59-fold, respectively (Figure 5). However, E2 inhibitor ICI reversed this antihypertrophy effect in NRMCs. These data suggest that the AMPK pathway plays important role in SIRT1-mediated cardiomyocyte protection.

We next examined the possibility that E2 suppressed cell apoptosis by regulating cell survival signals. The pretreatment of cardiomyocytes with E2 for 2 h resulted in profoundly reduced AngII-induced cell death. The level of active caspase-3 significantly decreased 49.51% in AngII + E2 group compared with AngII group (Figure 5). The direct correlation of the E2, SIRT1 expression, and cardiomyocyte viability during AngII-induced oxidative stress suggested that SIRT1 might play a crucial role in E2's protection effect of oxidative stress-induced cardiomyocyte injury. These results indicate that elevated SIRT1 levels by E2 might attenuate cardiomyocyte apoptosis in AngII-treated NRVMs.

## 5. Discussion

Heart hypertrophy is the common cause of heart failure and leads to severe oxidative stress, cardiomyocyte apoptosis, and function loss, resulting in poor prognosis. Recent studies have provided unequivocal evidence that both cardiomyocyte hypertrophy and apoptosis during heart hypertrophy contributed to heart remodeling, whereas the inhibition of cardiomyocyte hypertrophy and apoptosis substantially prevented the progress of heart hypertrophy and the development of heart failure [18–20].

SIRT1 is a ubiquitous NAD (+) dependent deacetylase that plays an important role in biological processes such as metabolism, stress response, and aging. SIRT1 expression is very high in cardiomyocytes and is associated with heart functions and response to the stresses [21–23]. However, the link between SIRT1 and heart remodeling is complex and is not fully understood. This study focuses specifically on the response of SIRT1 to AngII-induced cardiomyocyte remodeling, including cardiomyocyte hypertrophy, oxidative stress, and apoptosis.

SIRT1 is associated with protection against many stresses. Its expression is upregulated in calorie restriction (CR) [4], heart hypertrophy [24], and inflammation [25]. So SIRT1's upregulation in many kinds of stress is the automatic protection effect of the cell or body. In the oxidative stress condition, the cells get the oxidative stress first and cause the damage, and then cells try to upregulate SIRT1 expression to protect themselves from further injury. AngII could cause cardiomyocyte oxidative stress and induce moderate SIRT1 upregulation. But this moderate elevated level of SIRT1 was not sufficient to rescue the cell from oxidative stress and apoptosis. E2 is very efficient in regulating SIRT1 expression in the stress conditions. With the pretreatment of E2, the SIRT1 was upregulated with higher level, which could protect the cardiomyocyte from Ang II-induced oxidative stress.

Estrogen has been shown to provide cardioprotection in various models of cardiac disease [16, 17]. AngII-induced myocardial oxidative stress could lead to cardiomyocyte loss [26, 27]. AMPK is a serine/threonine enzyme that participates in the control of fundamental cellular processes such as growth, proliferation, and survival. The energy-sensing capability of AMPK is attributed to its ability to detect and react to fluctuations in the AMP: ATP ratio that take place during heart hypertrophy. In the present study, we demonstrated that E2 could promote AMPK phosphorylation and SIRT1 expression and protect cardiomyocyte function [3, 28, 29]. AngII could induce caspase-3 activation (cleaved caspase-3 form), while E2 and SIRT1 overexpression could block the activation of caspase-3 and promote cell survival. These findings indicate that estrogen and SIRT1 might play the same role in regulating cardiomyocyte function by inhibiting apoptosis. Moreover, SIRT1 active blocker niacinamide was able to increase ROS production and apoptosis following AngII stimulation, indicating that SIRT1 was essentially involved in E2-mediated heart protection by inhibiting the apoptosis signaling pathway in response to oxidative stress. Therefore, SIRT1 might attenuate AngII-induced cardiomyocyte death, highlighting the importance of SIRT1 in E2-mediated cardiac protective signaling.

Our results demonstrate that SIRT1 overexpression is sufficient to block AngII-induced cardiomyocyte hypertrophy and apoptosis in cultured NRVMs by regulating the expression of several important survival genes and pro-apoptotic genes. SIRT1 inhibitor experiments also indicated that SIRT1 is required for cardioprotection in AngII-induced heart remodeling. More importantly, SIRT1 blocker exaggerated AngII-induced oxidative stress and pro-apoptotic signal. Here, we identified SIRT1 as a potential genetic modifier and an important transcription factor attenuating cardiomyocyte hypertrophy and apoptosis.

Multiple lines of evidence suggest that SIRT1 was involved in E2-regulated heart protection. These findings define E2-SIRT1 as an important regulatory cascade in E2-mediated heart protection, thus providing a potentially important novel therapeutic target for the treatment of heart failure disease.

Previous studies have demonstrated that SIRT1 is required for heart metabolism and function. In the present study, we found that SIRT1 was slightly regulated in heart hypertrophy mouse model and AngII-treated NRVMs, indicating that SIRT1 might act as an inner protective regulator in hearts. It is particularly interesting that E2 could promote SIRT1 expression in stress conditions because clinical association studies revealed an association between E2 and heart failure; and overexpression of SIRT1 could block cell hypertrophy and apoptosis. These results demonstrate a pivotal role for SIRT1 in the precise regulation of cardiomyocyte metabolism, function, and survival. The dysregulation of SIRT1 may be a common feature of the development of heart hypertrophy and therefore represents a potential therapeutic target.

Before these findings can be applied to clinical medicine, several important issues must be addressed. First, although our study clearly indicates that SIRT1 is necessary for the E2-mediated cell protection, it awaits future investigation to determine how E2 regulates SIRT1 expression. This study primarily focused on SIRT1's antihypertrophy and antiapoptotic effects. Further studies are required to define the potential role of SIRT1 in heart remodeling.

## 6. Conclusions

These results indicate that SIRT1 functions as an important regulator of estrogen-mediated cardiomyocyte protection during Angiotensin II-induced heart hypertrophy and injury, suggesting that estrogen and SIRT1 may represent a crucial regulatory cascade and a potential therapeutic target for heart hypertrophy and injury.
